# Supplementary material for: A single-cell atlas of spatial and temporal gene expression in the mouse cranial neural plate
Source: bioRxiv. 2025 Mar 6:2024.08.25.609458. Originally published 2024 Aug 25. Preprint. [Version 3] doi: 10.1101/2024.08.25.609458 (PMC11370589; doi:10.1101/2024.08.25.609458)

Figure 1 - Figure Supplement 1

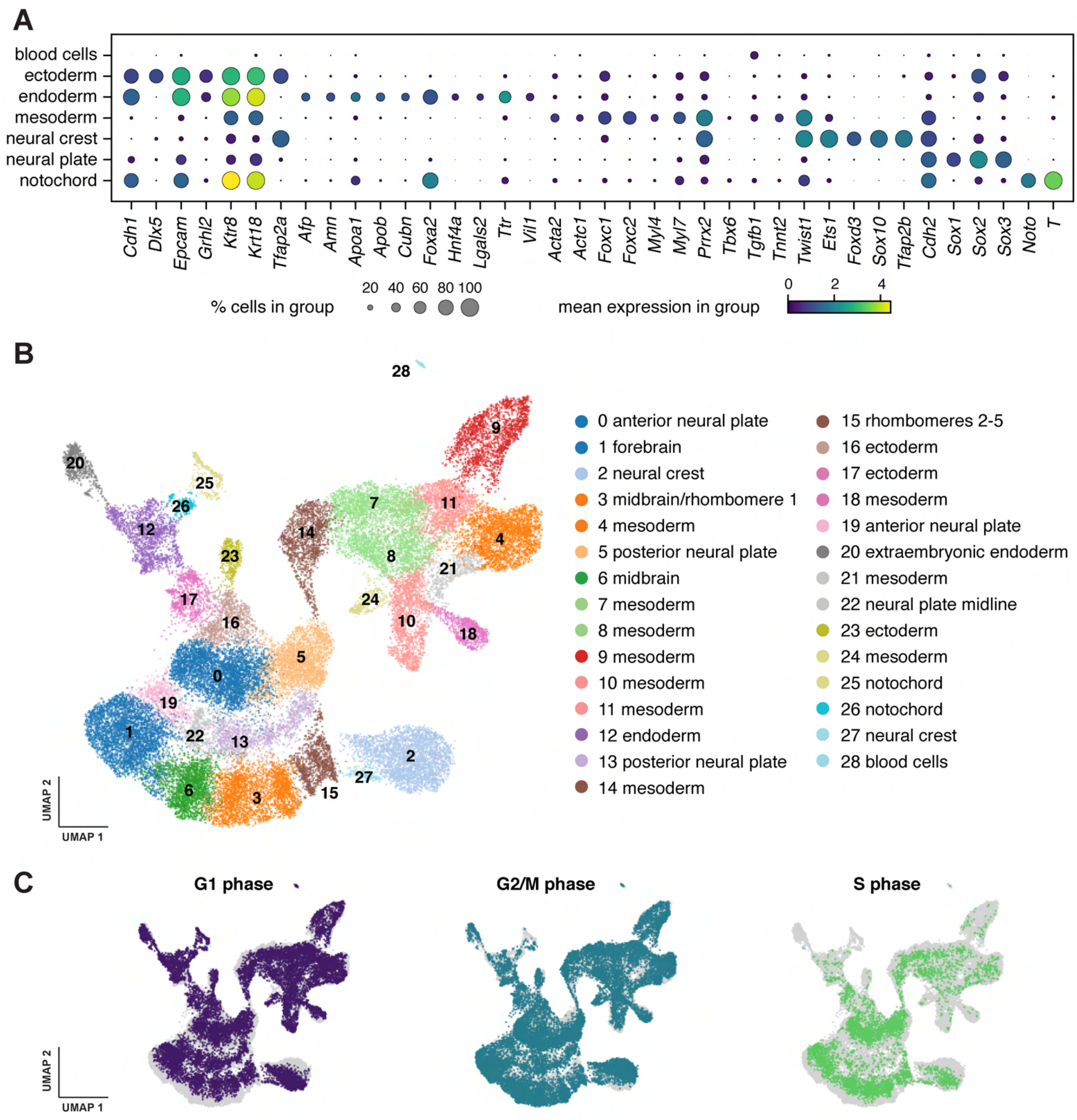

Figure 1 - Figure Supplement 2

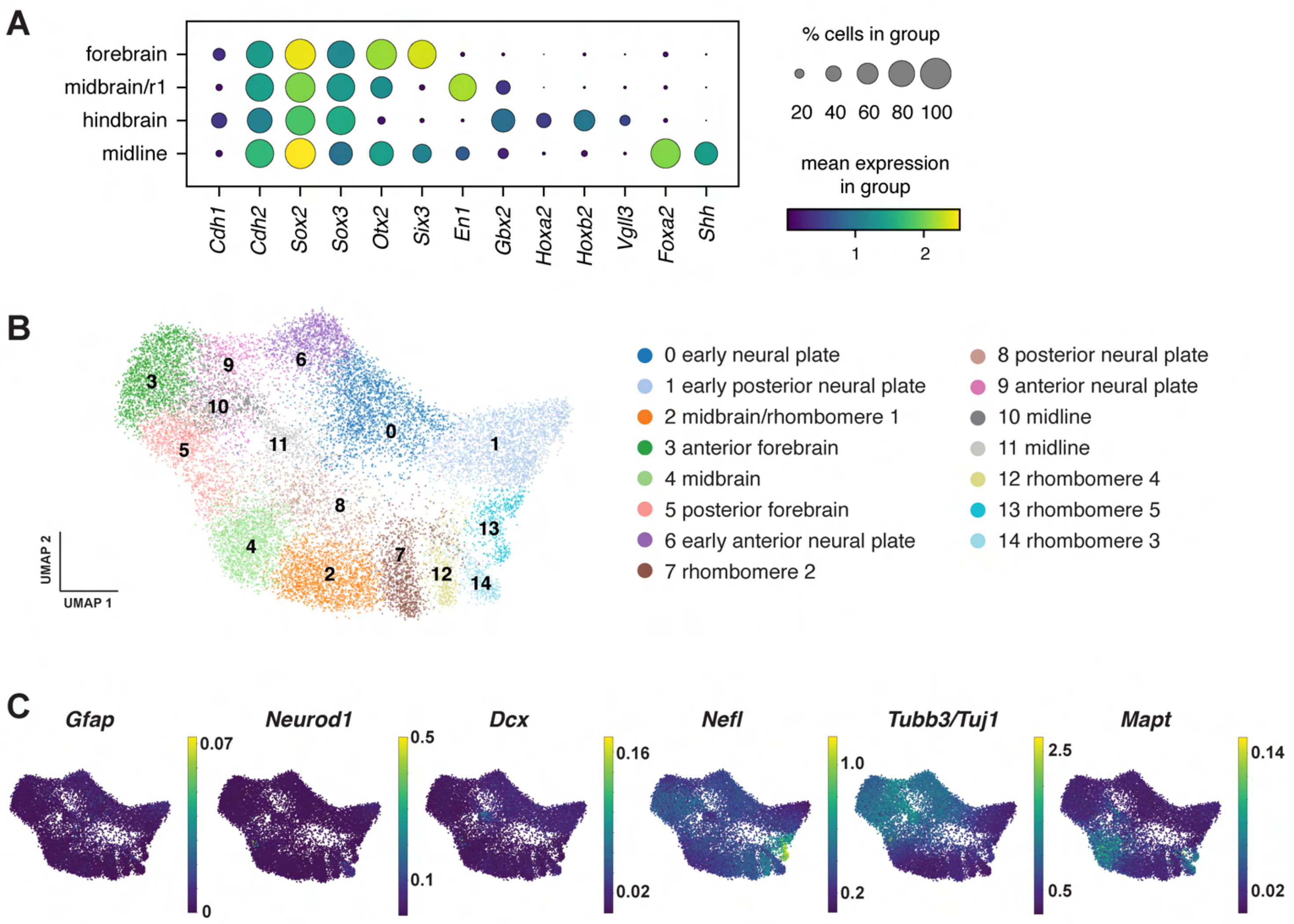

Figure 2 - Figure Supplement 1

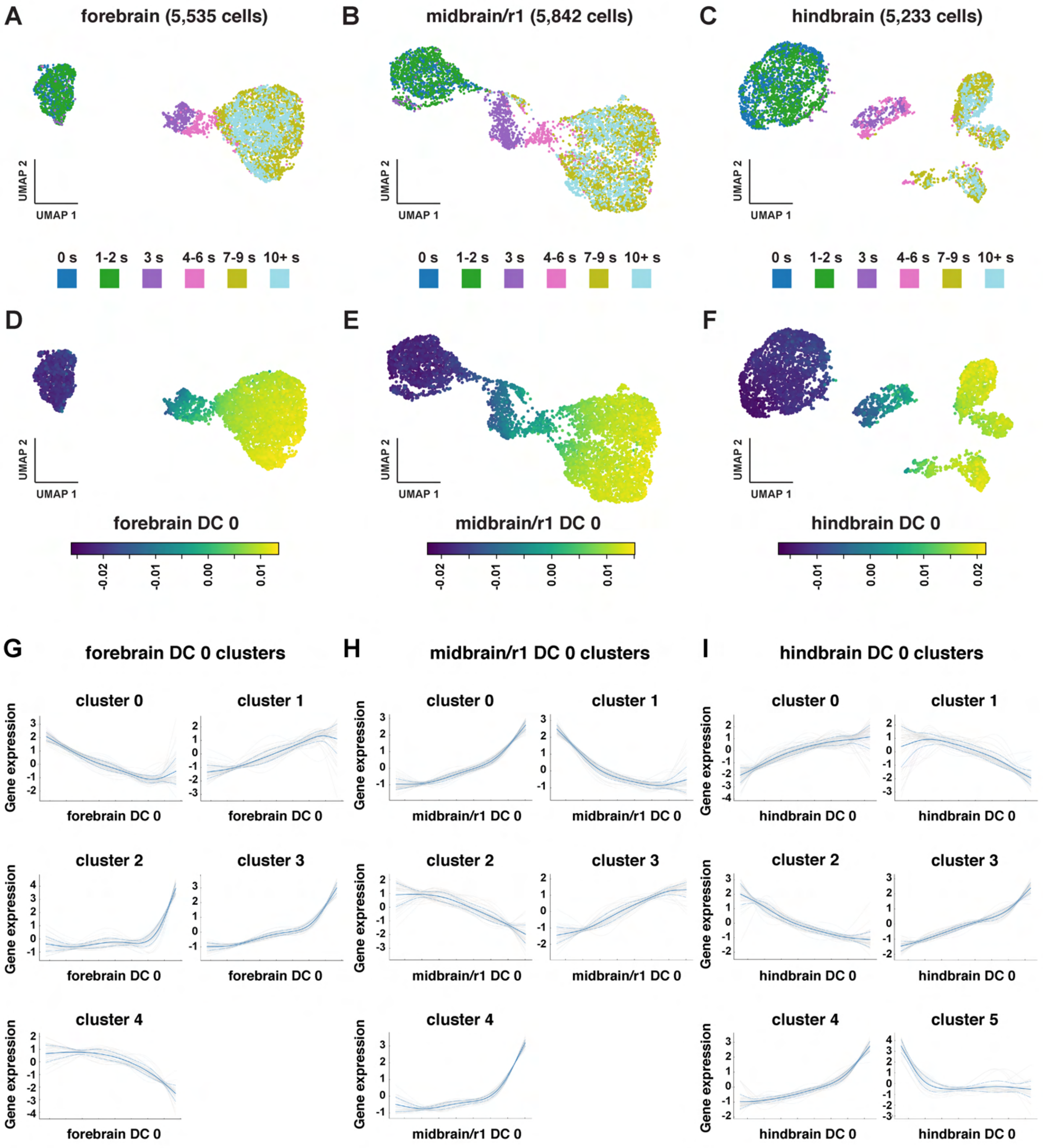

Figure 2 - Figure Supplement 2

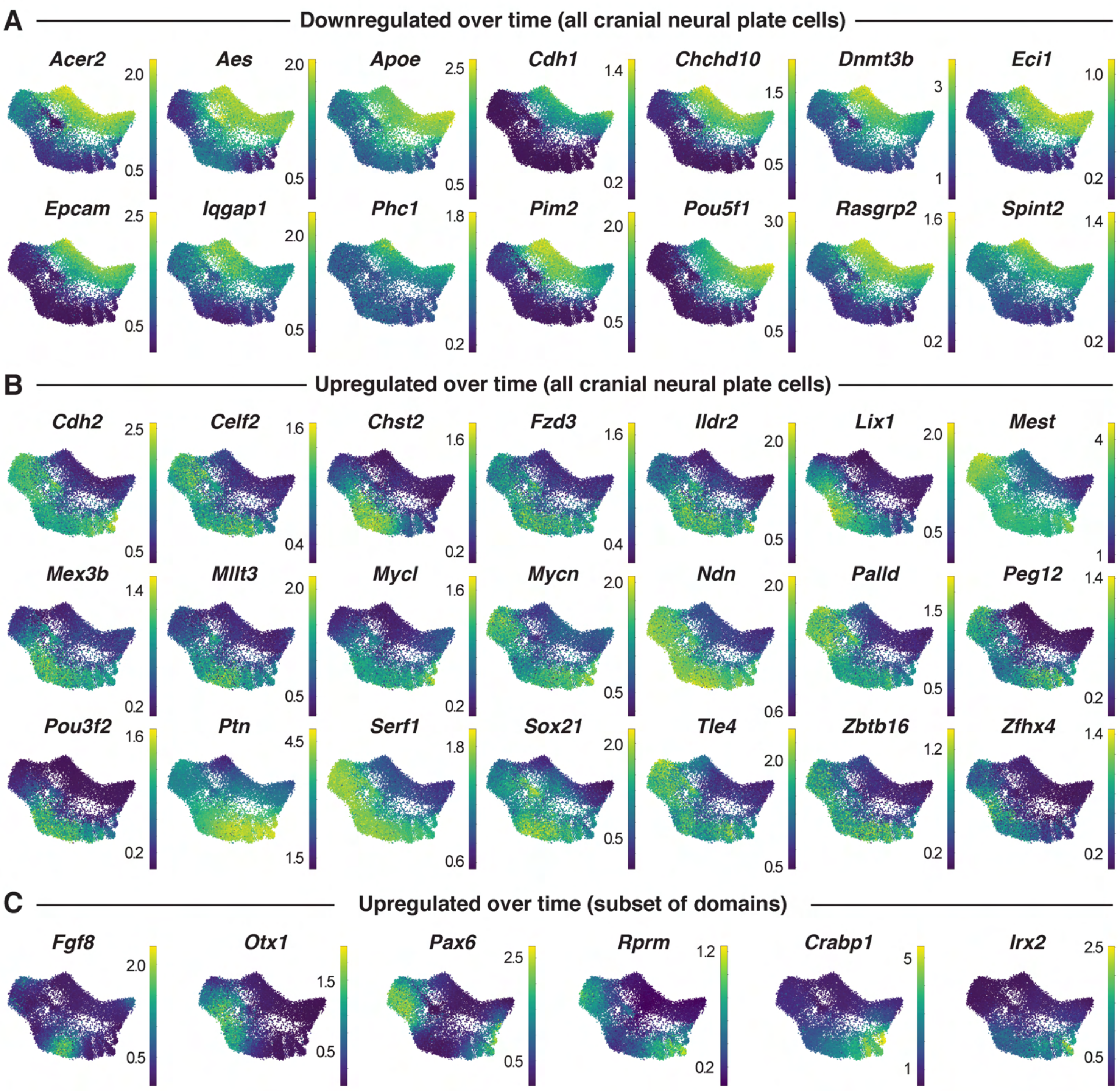

Figure 2 - Figure Supplement 3

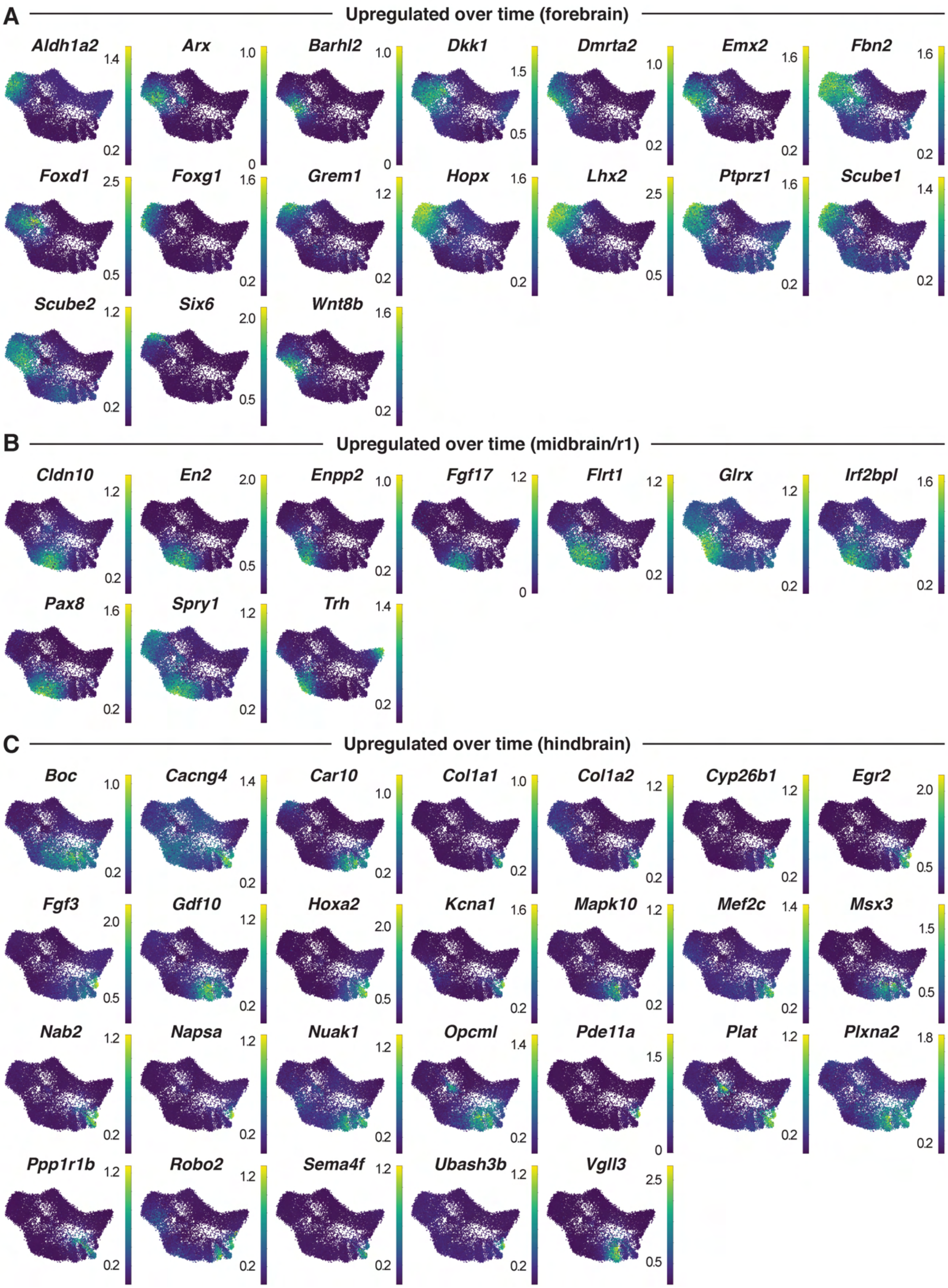

Figure 3 - Figure Supplement 1

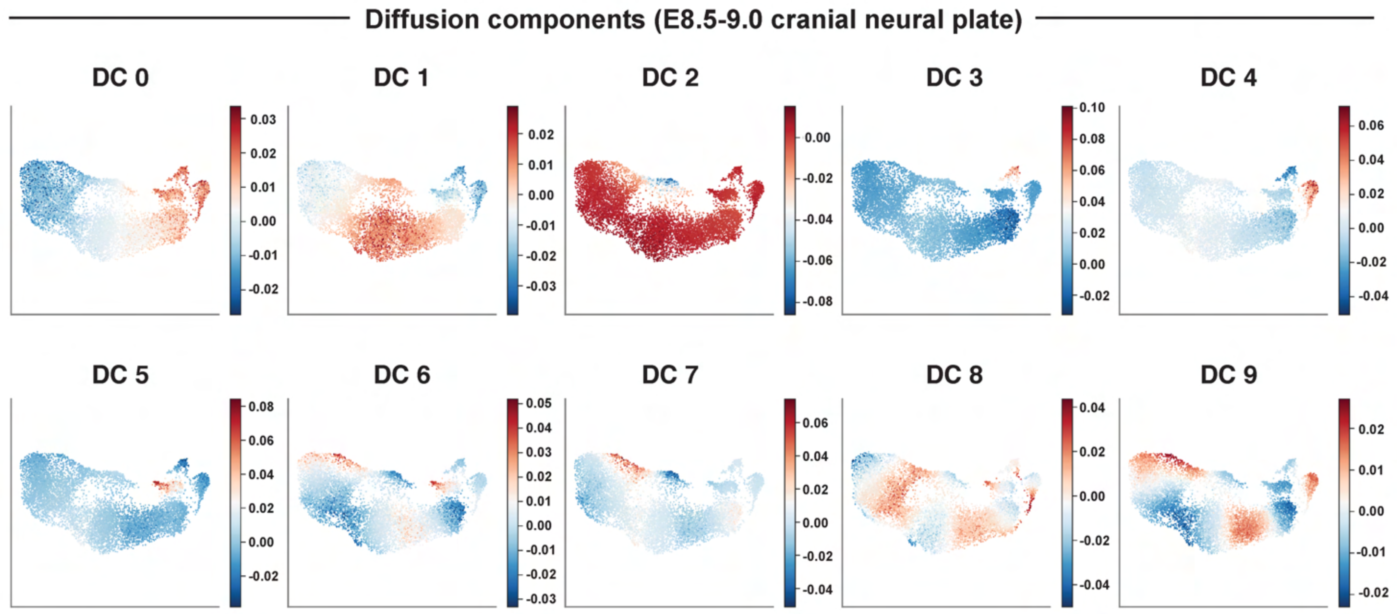

Figure 3 - Figure Supplement 2

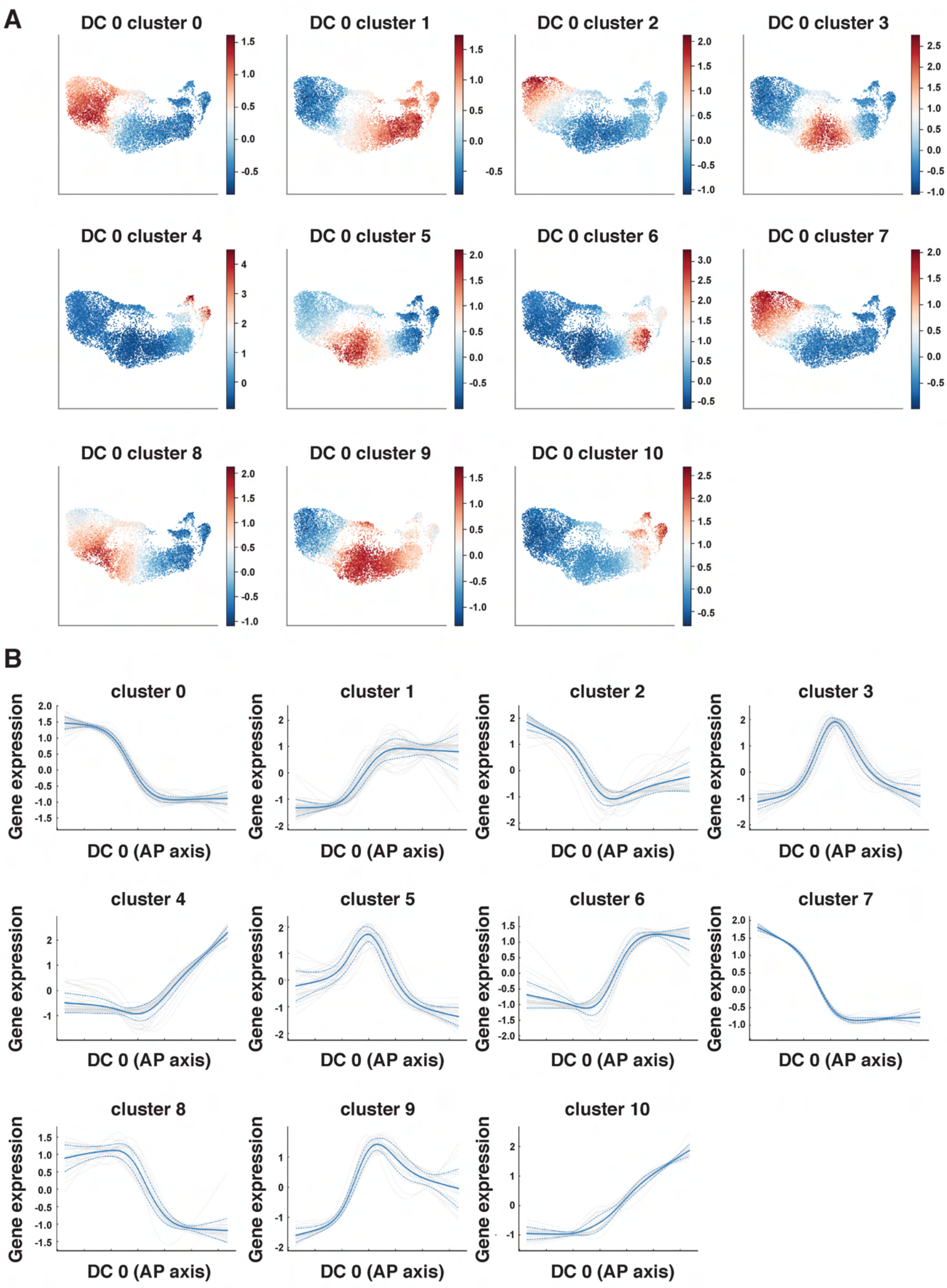

Figure 4 - Figure Supplement 1

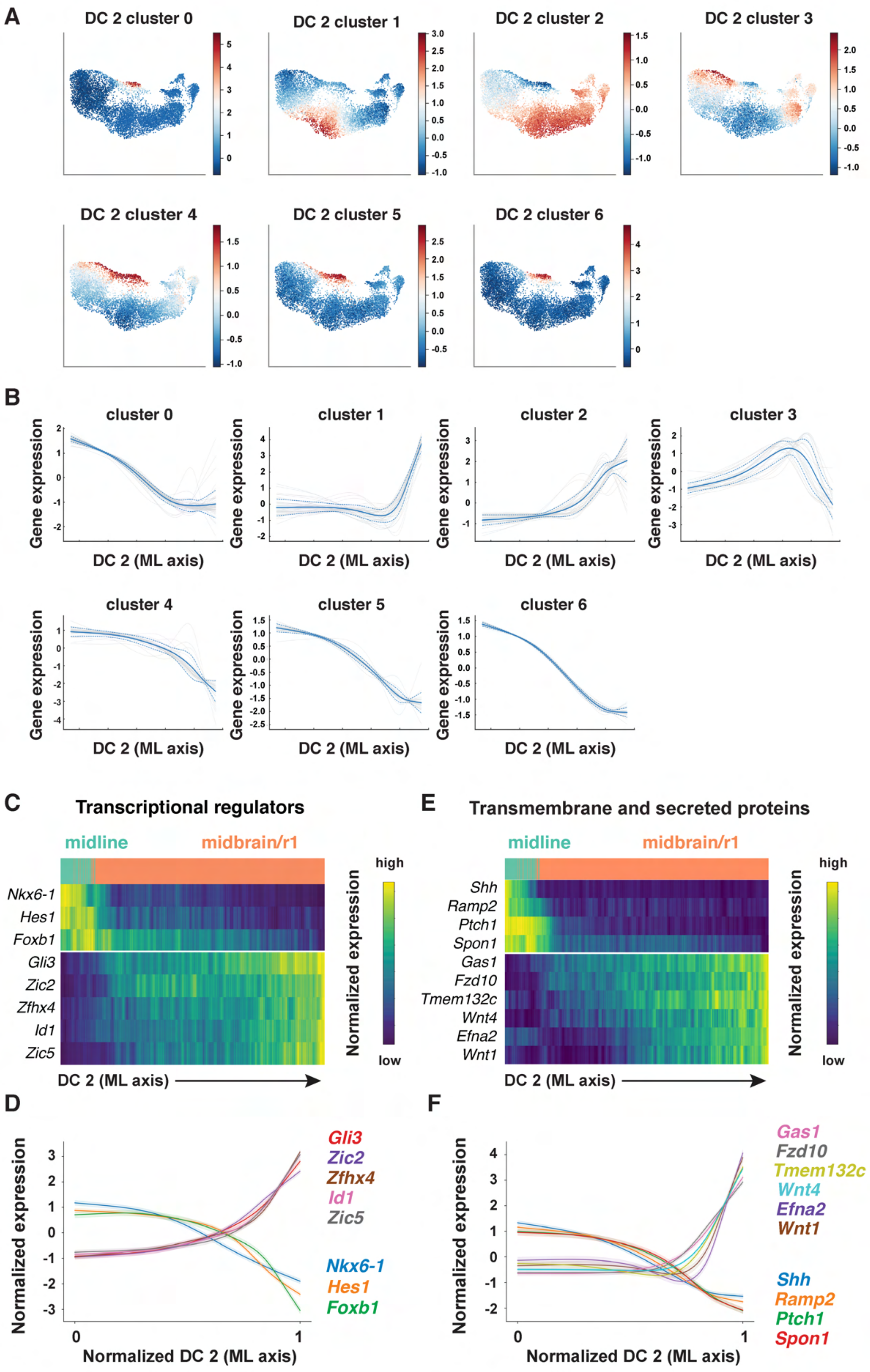

Figure 5 - Figure Supplement 1

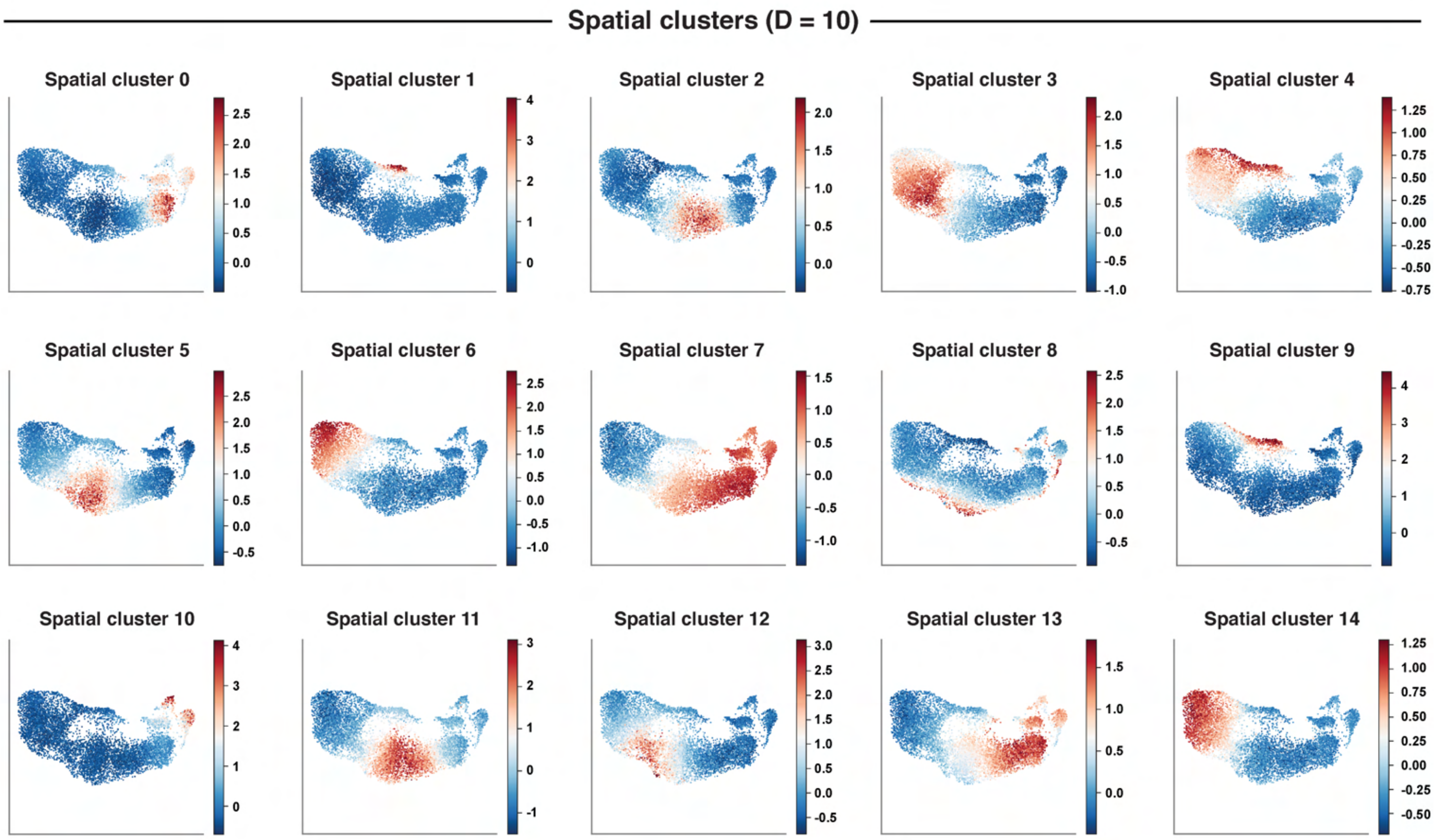

Figure 5 - Figure Supplement 2

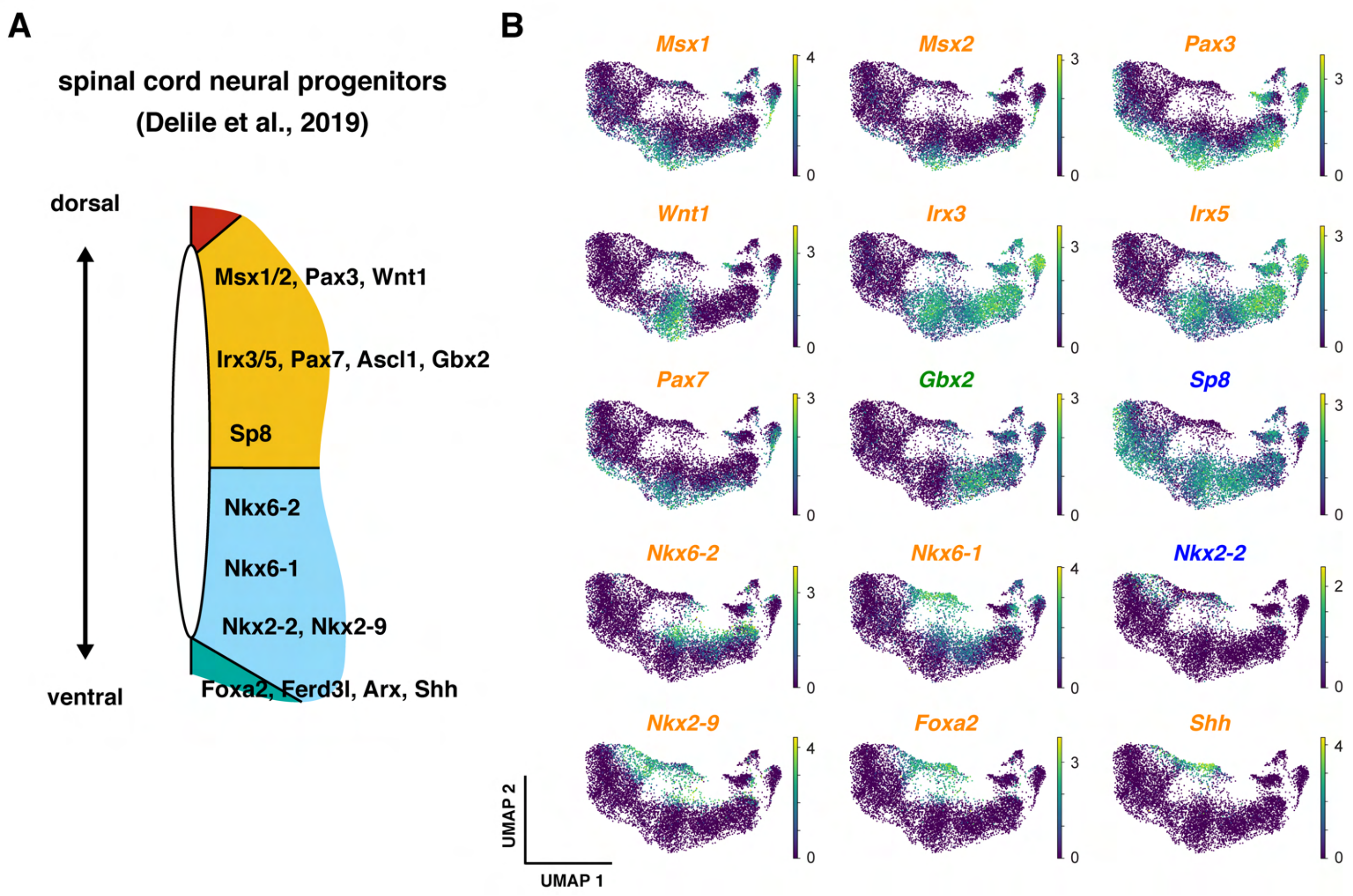

Figure 6 - Figure Supplement 1

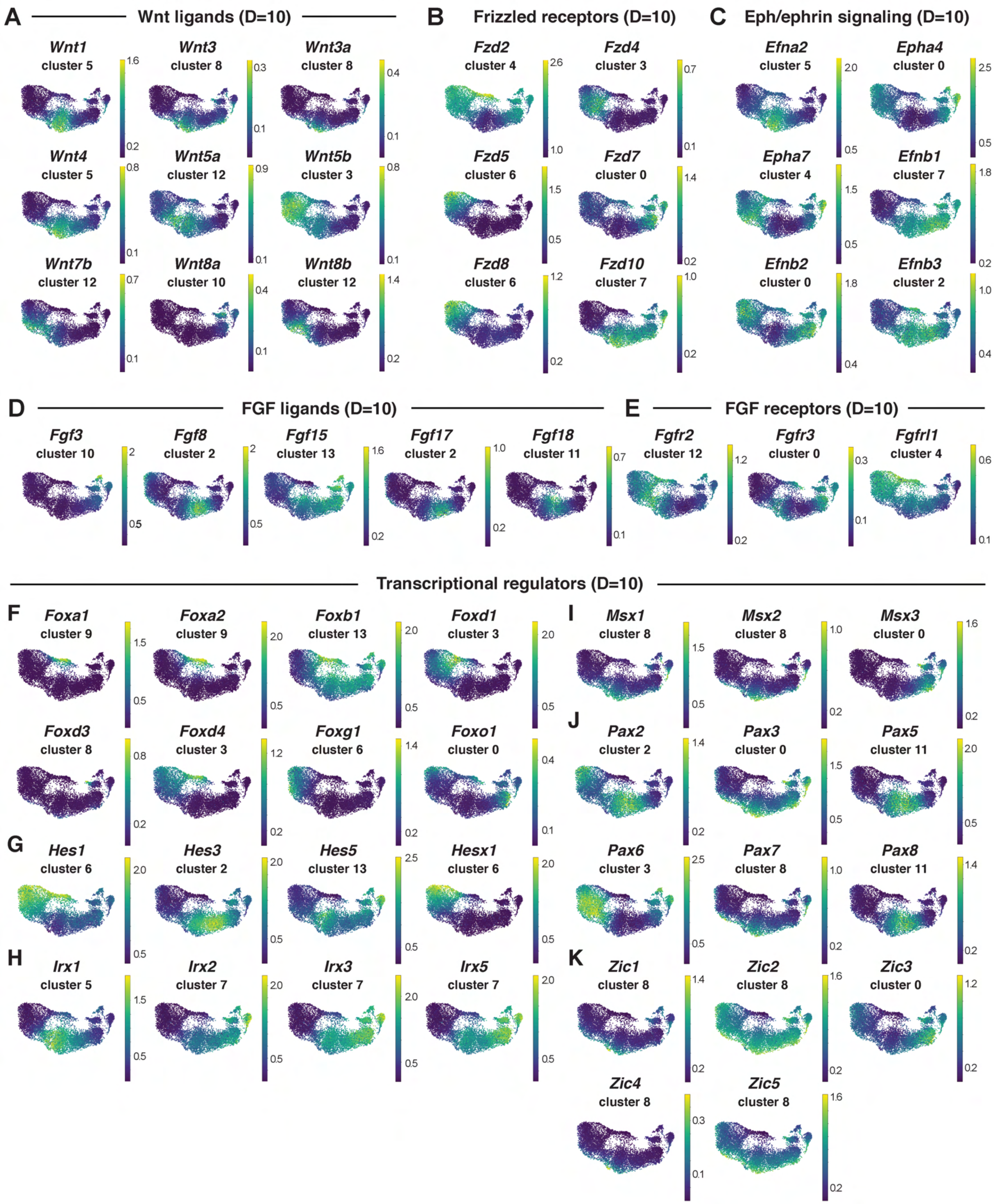

Supplement: Supplement 1 — Figure 1—figure supplement 1. Assignment of cell identities in the mouse cranial region. (A) Dot plot showing the normalized expression of a subset of markers used for cell type determination. (B) UMAP projection of all cranial cells analyzed (39,463 cells), colored by PhenoGraph cluster. Assigned cell types are listed on the right. (C) Distribution of cell cycle stages in the dataset. Figure 1—figure supplement 2. Assignment of cell identities in the mouse cranial neural plate. (A) Dot plot showing the normalized expression of a subset of markers used to assign cells to different neural plate regions. (B) UMAP projection of cranial neural plate cells (17,695 cells) reclustered in the absence of other cell types, colored by PhenoGraph cluster. Assigned neural plate regions are listed on the right. (C) UMAP projections of cranial neural plate cells colored by normalized gene expression. Markers for radial glial cells (Gfap) and immature neurons (Neurod1, Dcx) were not detected in the cranial neural plate at these stages, suggesting that cells are pre-neurogenic, although weak expression of neuronal cytoskeletal components (Nefl, Tubb3/Tuj1, Mapt) was observed. Figure 2—figure supplement 1. Analysis of gene expression trends in the developing forebrain, midbrain/r1, and hindbrain. (A-F) UMAP projections of neural plate cells separated by region based on known markers of anterior-posterior identity. Cells are colored by embryo stage (A-C) or by their value along the top diffusion component (DC0) for each region (D-F). Higher DC0 values correlate with later time points. (G-I) Gene expression profiles were clustered by differential expression along DC0 and the average normalized expression (solid blue line) +/− 1 standard deviation (dotted blue lines) and the expression of all individual genes in each cluster (gray lines) are shown. Figure 2—figure supplement 2. Examples of genes that are temporally regulated throughout the cranial neural plate. (A-C) UMAP projections of [file media-1.pdf]
