# Supplementary material for: A single-cell atlas of spatial and temporal gene expression in the mouse cranial neural plate
Source: bioRxiv. 2024 Aug 25:2024.08.25.609458. Preprint. [Version 1] doi: 10.1101/2024.08.25.609458 (PMC11370589; doi:10.1101/2024.08.25.609458)
Supplement: Supplementary file 1 [file NIHPP2024.08.25.609458v1-supplement-15.pdf]

bioRxiv preprint doi: https://doi.org/10.1101/2024.08.25.609458; this version posted August 26, 2024. The copyright holder for this preprint (which was not certified by peer review) is the author/funder, who has granted bioRxiv a license to display the preprint in perpetuity. It is made available under aCC-BY 4.0 International license.

Supplementary figure legends

Figure 1—figure supplement 1. Assignment of cell identities in the mouse cranial region. (A) Dot plot showing the normalized expression of a subset of markers used for cell type determination. (B) UMAP projection of all cranial cells analyzed (39,463 cells), colored by PhenoGraph cluster. Assigned cell types are listed on the right. (C) Distribution of cell cycle stages in the dataset.

Figure 1—figure supplement 2. Assignment of cell identities in the mouse cranial neural plate. (A) Dot plot showing the normalized expression of a subset of markers used to assign cells to different neural plate regions. (B) UMAP projection of cranial neural plate cells (17,695 cells) reclustered in the absence of other cell types, colored by PhenoGraph cluster. Assigned neural plate regions are listed on the right. (C) UMAP projections of cranial neural plate cells colored by normalized gene expression. Markers for radial glial cells (*Gfap*) and immature neurons (*Neurod1, Dcx*) were not detected in the cranial neural plate at these stages, suggesting that cells are pre-neurogenic, although weak expression of neuronal cytoskeletal components (*Nefl, Tubb3/Tuj1, Mapt*) was observed.

Figure 2—figure supplement 1. Analysis of gene expression trends in the developing forebrain, midbrain, and hindbrain. (A-F) UMAP projections of neural plate cells separated by region based on known markers of anterior-posterior identity. Cells are colored by embryo stage (A-C) or by their value along the top diffusion component (DC0) for each region (D-F). Higher DC0 values correlate with later time points. (G-I) Gene expression profiles were clustered by differential expression along DC0 and the average normalized expression (solid blue line) +/- 1 standard deviation (dotted blue lines) and the expression of all individual genes in each cluster (gray lines) are shown.

Figure 2—figure supplement 2. Examples of genes that are temporally regulated throughout the cranial neural plate. (A-C) UMAP projections of cranial neural plate cells colored

bioRxiv preprint doi: https://doi.org/10.1101/2024.08.25.609458; this version posted August 26, 2024. The copyright holder for this preprint (which was not certified by peer review) is the author/funder, who has granted bioRxiv a license to display the preprint in perpetuity. It is made available under aCC-BY 4.0 International license.

by normalized gene expression. Genes that display globally decreasing expression (A), globally increasing expression (B), or increasing expression in a subset of domains (C) are shown.

Figure 2—figure supplement 3. Examples of genes that are upregulated in the forebrain, midbrain, or hindbrain. (A-C) UMAP projections of cranial neural plate cells colored by normalized gene expression. Genes that display increasing expression in the future forebrain (A), midbrain/MHB (B), or hindbrain (C) are shown.

Figure 3—figure supplement 1. Diffusion component analysis of E8.5-9.0 neural plate cells. UMAP projections of neural plate cells from E8.5-E 9.0 embryos colored by the value along the indicated diffusion component. Red indicates higher DC values and blue indicates lower DC values. The top 10 diffusion components calculated for the dataset are shown.

Figure 3—figure supplement 2. Clustering analysis reveals distinct patterns of gene expression along DC0 in the E8.5-9.0 cranial neural plate. (A and B) Gene expression profiles in the E8.5-9.0 neural plate were clustered by differential expression along DC0, which correlates with cell position along the anterior-posterior axis. (A) UMAP projections of E8.5-9.0 neural plate cells colored by the average gene expression for each cluster. Red indicates higher expression and blue indicates lower expression of the genes in each cluster. (B) The average normalized gene expression for each cluster (solid blue line) +/- 1 standard deviation (dotted blue lines) and all individual genes in each cluster (gray lines) are shown.

Figure 4—figure supplement 1. Clustering analysis reveals distinct patterns of gene expression along DC2 in the E8.5-9.0 cranial neural plate. (A and B) Gene expression profiles in the E8.5-9.0 neural plate were clustered by differential expression along DC2, which correlates with cell position along the mediolateral axis. (A) UMAP projections of E8.5-9.0 neural plate cells colored by the average gene expression for each cluster. Red indicates higher expression and blue indicates lower expression of genes in the cluster. (B) The average normalized gene expression for each cluster (solid blue line) +/- 1 standard deviation (dotted blue lines) and all individual genes in each cluster (gray lines) are shown. (C-F) Heatmaps (C and E) and line plots (D and F) showing the normalized expression of a subset of transcriptional regulators (C and D) or transmembrane and secreted proteins (E and F) that appear to show graded changes in expression relative to DC2. Heatmaps show one gene per row, one cell per column, with the cells in each row ordered by their value along DC2. Cells assigned to the midline or midbrain/MHB are indicated at the top of each heatmap.

Figure 5—figure supplement 1. Multiscale clustering analysis reveals spatial patterns of gene expression in the cranial neural plate. UMAP projections of E8.5-9.0 neural plate cells colored by the average expression of genes in each spatial cluster. Red indicates higher expression and blue indicates lower expression of the genes in each cluster.

Figure 6—figure supplement 1. Patterned expression of gene families. (A-K) UMAP projections of E8.5-9.0 neural plate cells colored according to relative expression of an individual member of the indicated gene family. (A) *Wnt* ligands, (B) *Frizzled* receptors, (C) *Eph/Ephrin*

bioRxiv preprint doi: https://doi.org/10.1101/2024.08.25.609458; this version posted August 26, 2024. The copyright holder for this preprint (which was not certified by peer review) is the author/funder, who has granted bioRxiv a license to display the preprint in perpetuity. It is made available under aCC-BY 4.0 International license.

signaling molecules, (D) *Fgf* ligands, (E) *Fgf* receptors, (F) *Fox* transcription factors, (G) *Hes* transcription factors, (H) *Irx* transcription factors, (I) *Msx* transcription factors, (J) *Pax* transcription factors, and (K) *Zic* transcription factors. Spatial cluster identities at a stringency of D=10 are shown.

Table legends

Table S1. Cell type and quality control metrics for wild-type and SAG treatment datasets. The total number of cells post-filtering, the number of cells assigned to each cell type, and quality metrics (median number of UMIs, median number of genes, median percent mitochondrial UMIs, median percent ribosomal UMIs, and mean reads/UMI) are shown for each replicate in the wild-type, SAG treatment, and SAG control datasets.

Table S2. PhenoGraph clustering of all cranial cells in E7.5-9.0 embryos. Gene expression for each PhenoGraph cluster in the full cranial cell dataset. The false-discovery rate (FDR) adjusted p-value, MAST hurdle value, and empirical $\log_2$ fold change are shown for each gene.

Table S3. PhenoGraph clustering of cranial neural plate cells in E7.5-9.0 embryos. Gene expression for each PhenoGraph cluster in the cranial neural plate are shown. The false-discovery rate (FDR) adjusted p-value, MAST hurdle value, and empirical $\log_2$ fold change are shown for each gene.

Table S4. Temporally regulated genes in the forebrain, midbrain, and hindbrain of E7.5-9.0 embryos. Gene correlation scores for the temporally correlated diffusion component (DC0) in each region are shown for the forebrain, midbrain/midbrain-hindbrain boundary, and hindbrain cranial neural plate populations. The cluster number, Pearson correlation coefficient, and normalized range of expression are shown for each gene.

Table S5. Diffusion component gene correlations in the E8.5-9.0 cranial neural plate. Gene correlations with each of the top 10 diffusion components in the E8.5-9.0 cranial neural plate are shown. The false discovery rate (FDR) adjusted p-value and Pearson correlation coefficient for each diffusion component are shown for each gene.

Table S6. Spatially patterned genes in the E8.5-9.0 cranial neural plate. All genes detected in cells from wild-type E8.5-9.0 embryos are shown. The results of HotSpot analysis are given, including the Geary's C spatial autocorrelation coefficient (C, MSD), the z-scored coefficient (Z, MSD), the p-value (Pval, MSD), the false discovery rate (FDR, MSD), and the normalized range of expression for each gene, in addition to the results of HotSpot analysis for the anterior-posterior (AP) axis-correlated diffusion component (DC0) and the mediolateral (ML) axis-correlated diffusion component (DC2). Cluster identities along the AP axis (DC0), ML axis (DC2), and at various 2D distance (D) values are shown.

Table S7. Comparison of predicted gene expression patterns to published data. For genes predicted to be spatially patterned in Table S6, the predicted expression of genes in anterior-

bioRxiv preprint doi: https://doi.org/10.1101/2024.08.25.609458; this version posted August 26, 2024. The copyright holder for this preprint (which was not certified by peer review) is the author/funder, who has granted bioRxiv a license to display the preprint in perpetuity. It is made available under aCC-BY 4.0 International license.

posterior (AP) or mediolateral (ML) clusters was compared with published images in the Mouse Genome Informatics Gene eXpression Database (GXD). In addition to the information in Table S6, the predicted and observed expression patterns and whether they agree is reported and a link to available GXD images of wild-type embryos from Theiler stages 12-14 is provided.

Table S8. Spatially patterned transcriptional regulators in the E8.5-9.0 cranial neural plate. Spatial (2D) cluster (distance D=10), AP cluster, and ML cluster of transcriptional regulators in the E8.5-9.0 cranial neural plate are shown.

Table S9. Spatially patterned secreted and transmembrane proteins in the E8.5-9.0 cranial neural plate. Spatial (2D) cluster (distance D=10), AP cluster, and ML cluster of secreted (ligands) and transmembrane (receptors) proteins in the dataset are shown.

Table S10. Differentially expressed genes in SAG-treated and control embryos in the E8.5 cranial neural plate. Results of MAST differential gene expression analysis in SAG-treated vs, control embryos separated by forebrain, midbrain/MHB, and hindbrain regions. The false discovery rate (FDR) adjusted p-value, MAST hurdle value, and empirical $\log_2$ fold change value are shown for each gene.

Table S11. Regional analysis of transcriptional changes upon SAG treatment. Genes with significant expression changes from Table S10 (FDR-adjusted p-value $p<0.001$) grouped by region, including all cranial neural plate regions (FB-MB-HB), two regions (FB-MB, FB-HB, MB-HB), or single regions (FB, MB, HB). Upregulated genes with a MAST hurdle value > 0.24 are highlighted in green. Downregulated genes with a MAST hurdle value < -0.24 are highlighted in red.

Table S12. Genes used for cell cycle normalization. Gene ontology (GO) terms associated with the cell cycle and the associated genes used in cell cycle normalization are listed.

Table S13. Genes used for cell typing. Genes used to assign cell/tissue type are listed, along with their positive or negative expression in that tissue and any expression overlap in other tissues.
