# Supplementary material for: A single-cell atlas of spatial and temporal gene expression in the mouse cranial neural plate
Source: bioRxiv. 2024 Aug 25:2024.08.25.609458. Preprint. [Version 1] doi: 10.1101/2024.08.25.609458 (PMC11370589; doi:10.1101/2024.08.25.609458)

Figure 1 - Figure Supplement 1

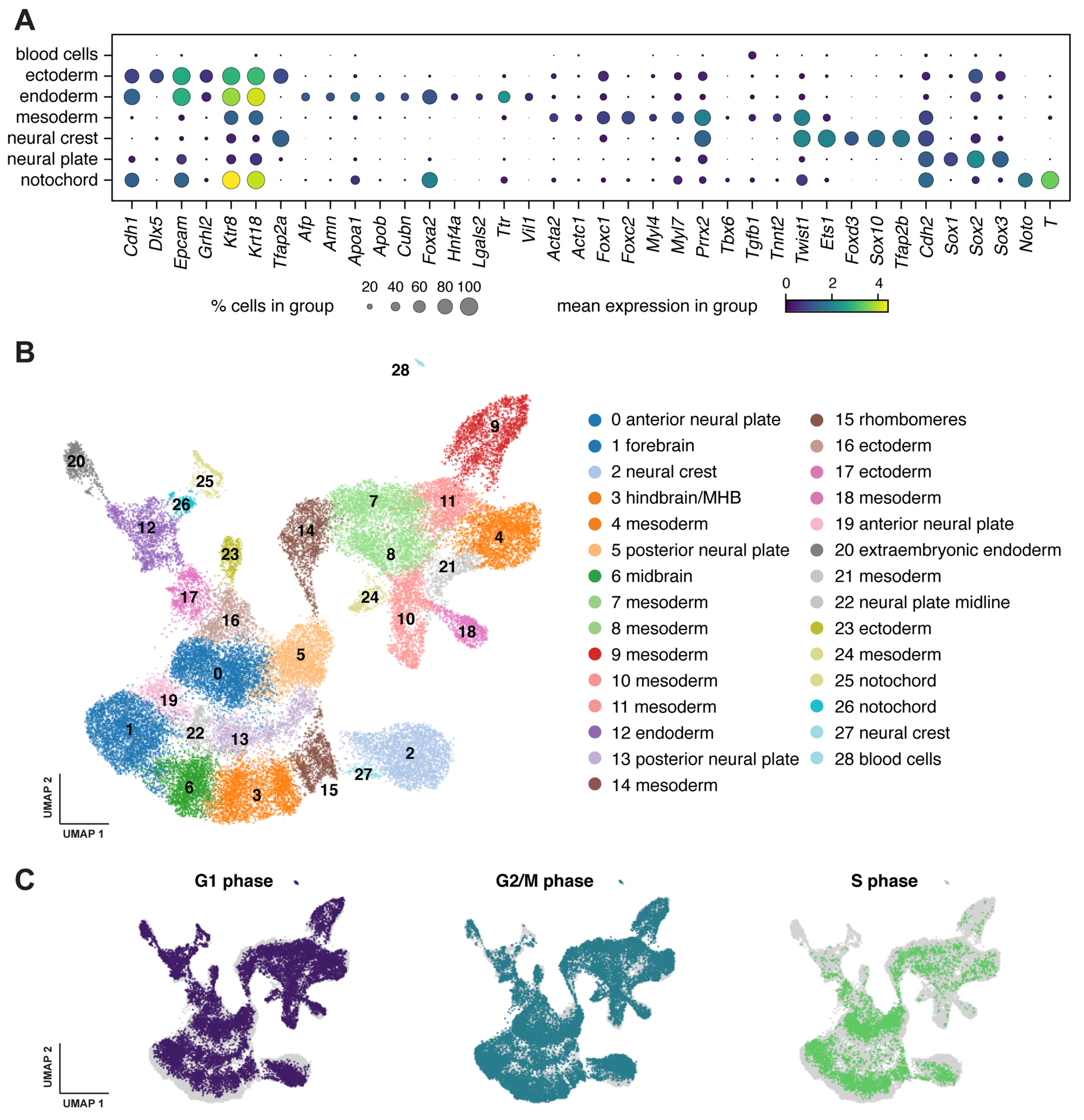

Figure 1 - Figure Supplement 2

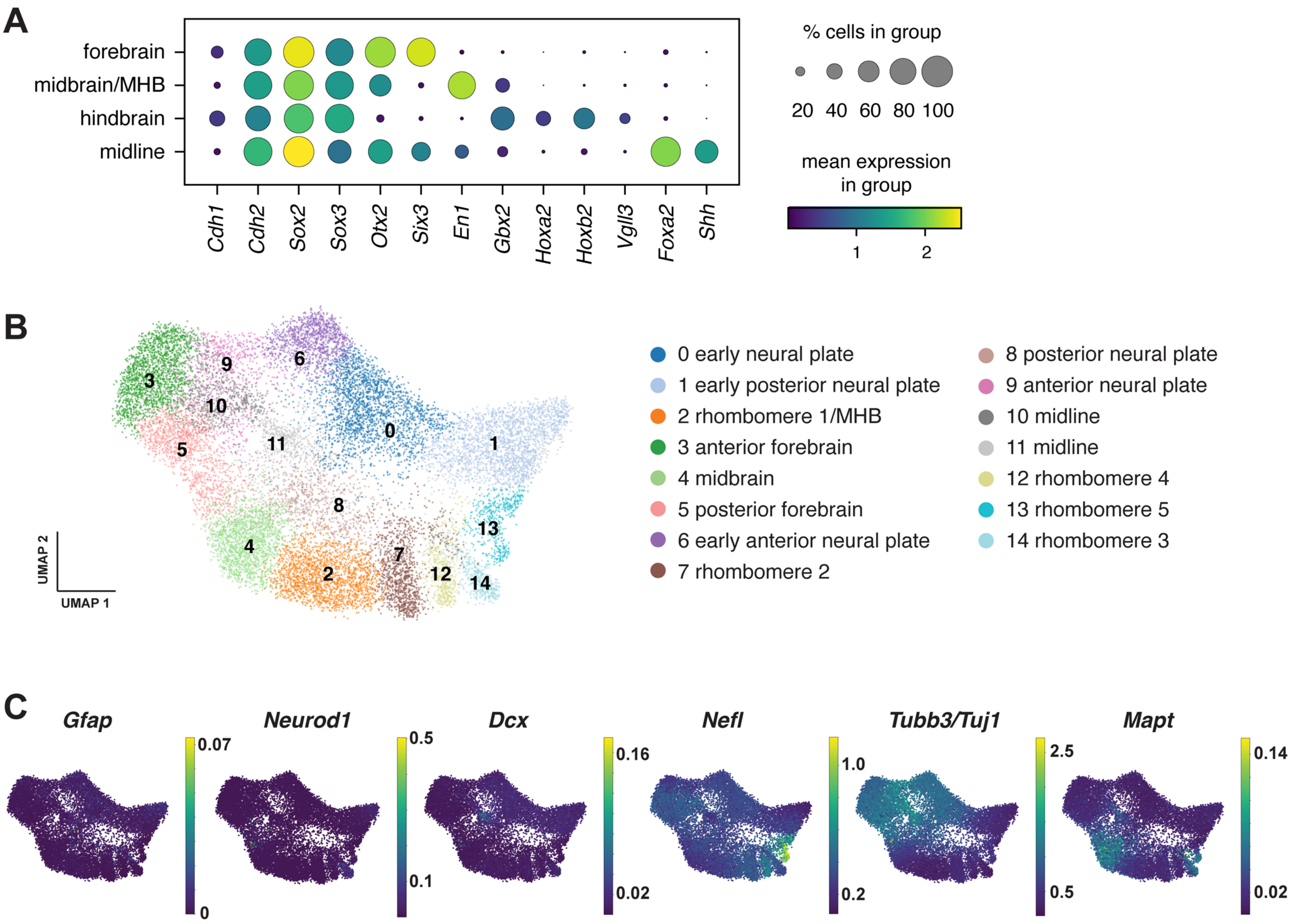

Figure 2 - Figure Supplement 1

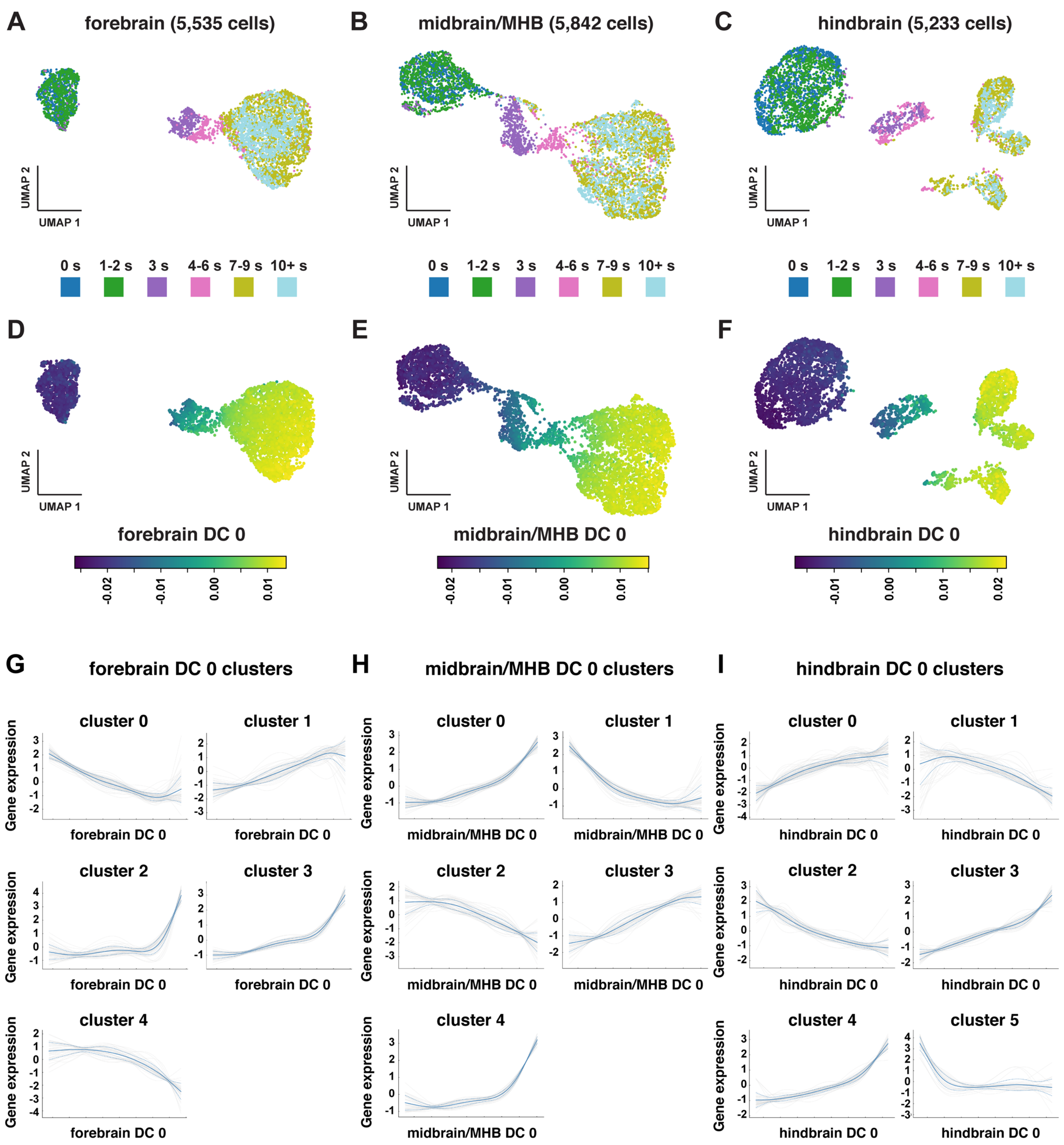

Figure 2 - Figure Supplement 2

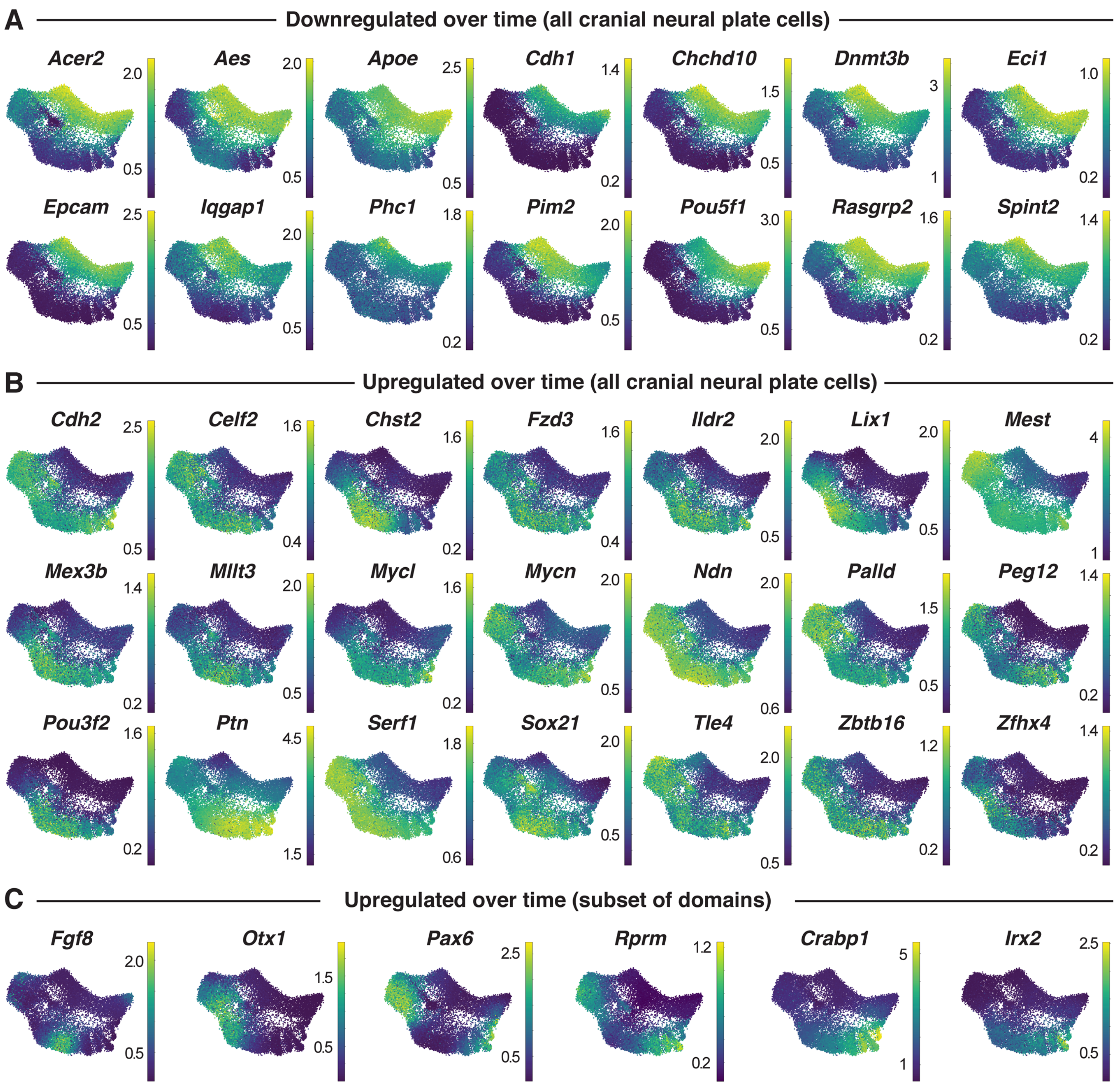

Figure 2 - Figure Supplement 3

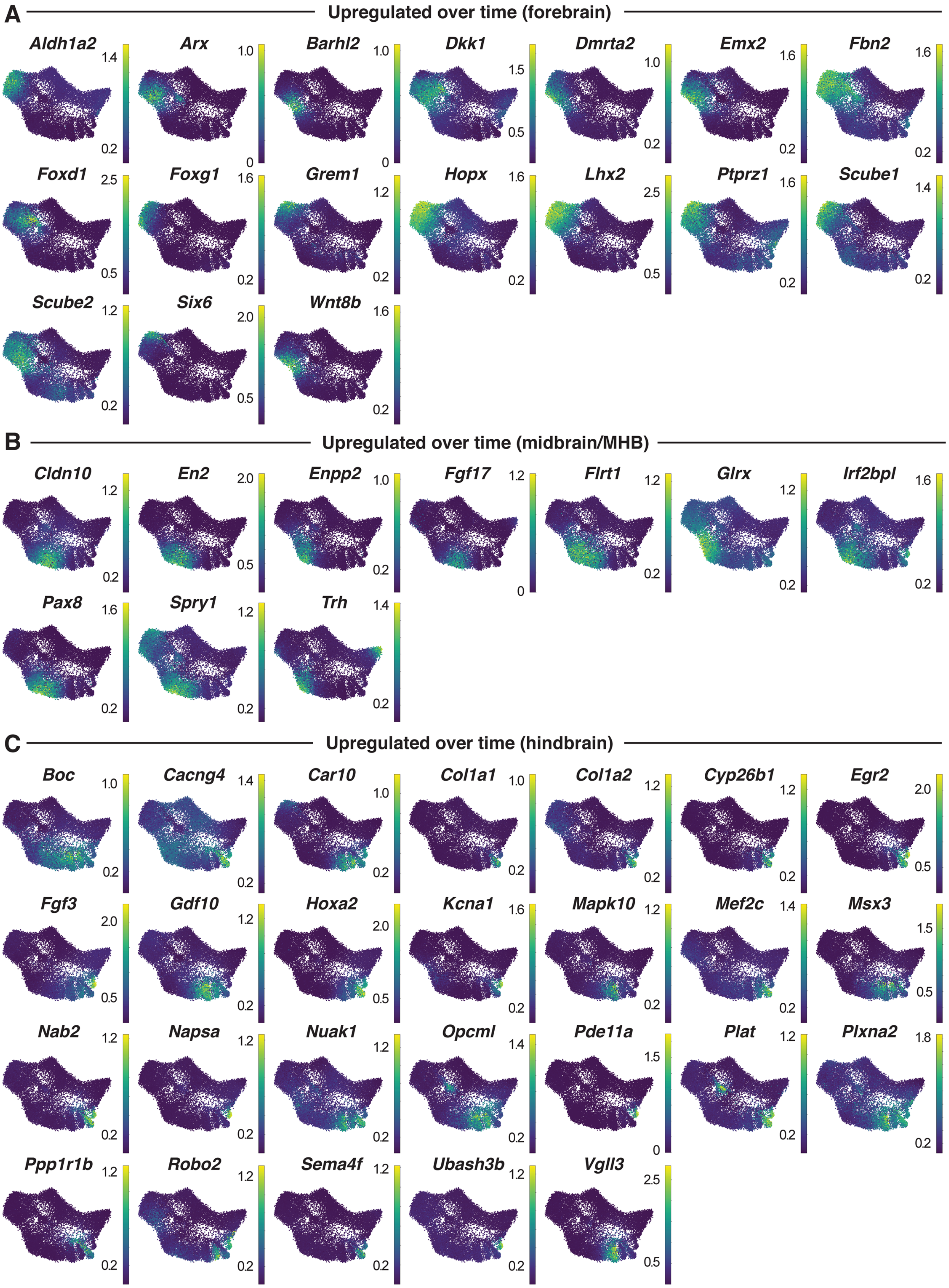

Figure 3 - Figure Supplement 1

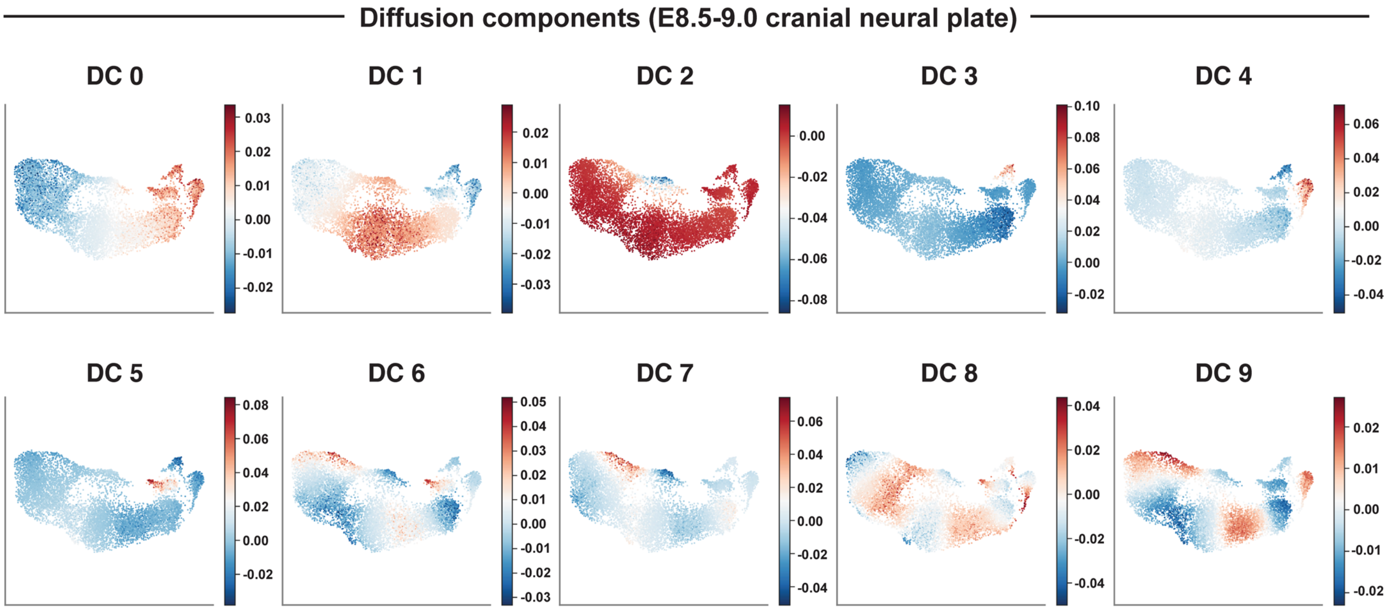

Figure 3 - Figure Supplement 2

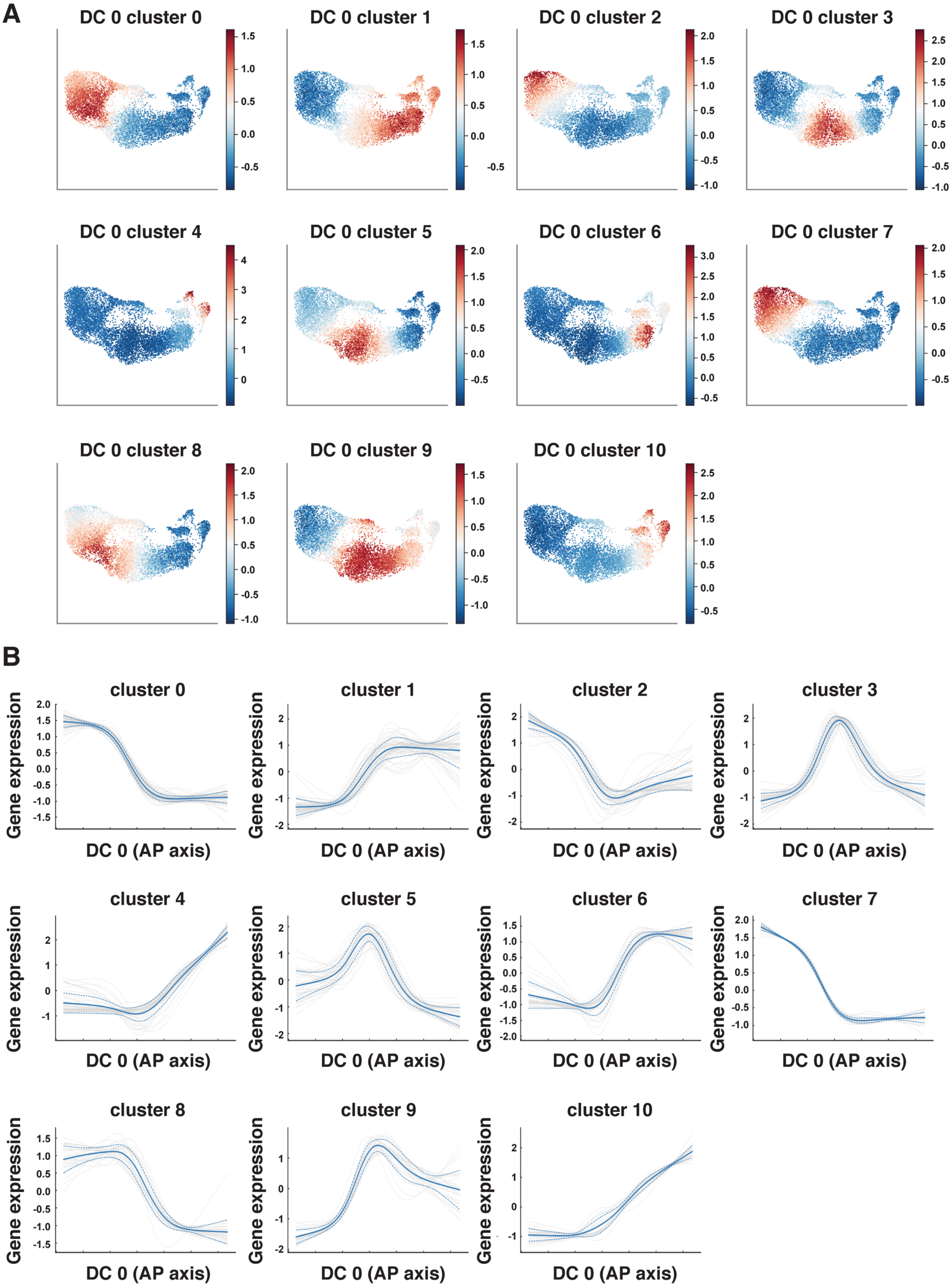

Figure 4 - Figure Supplement 1

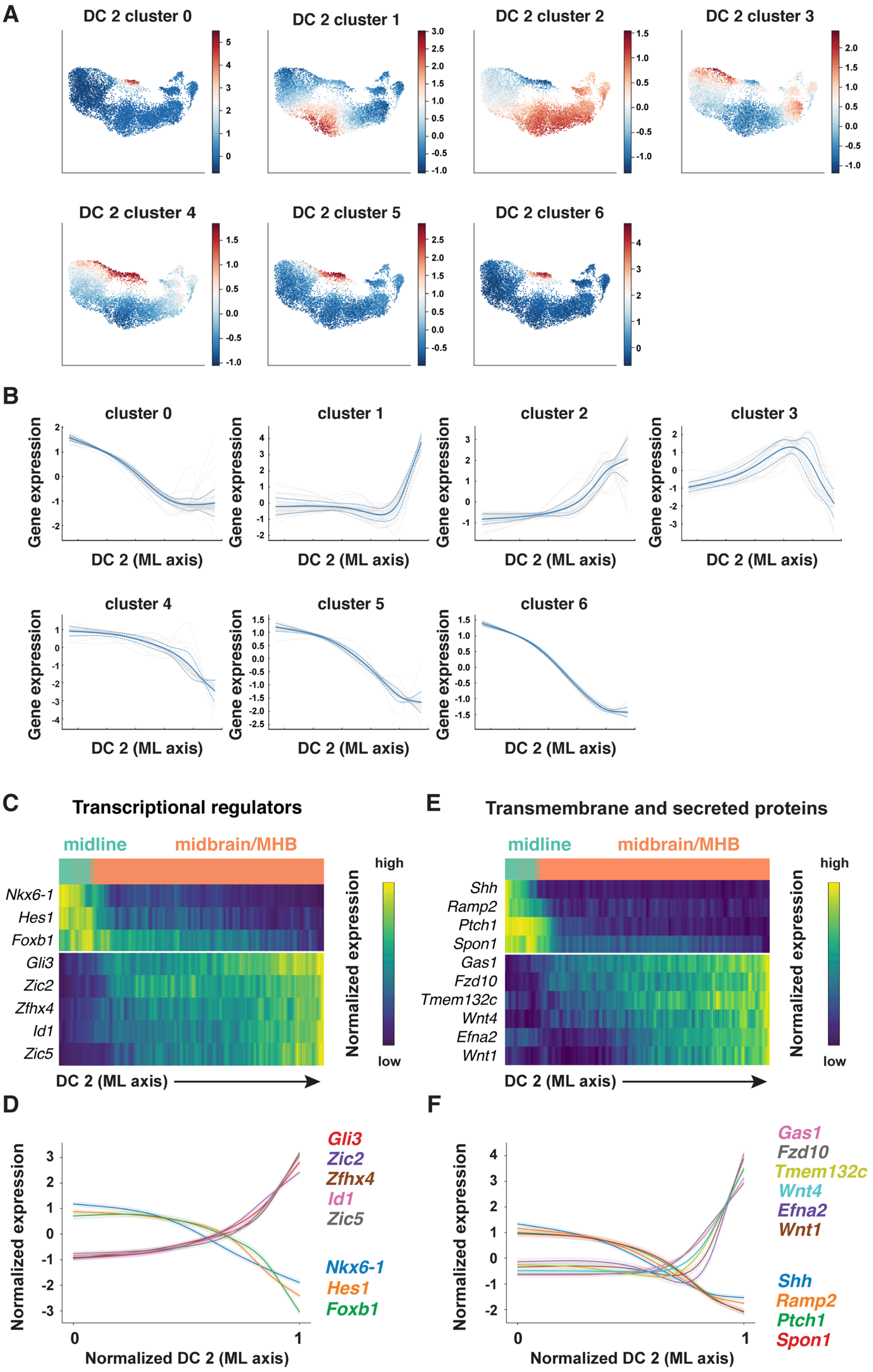

Figure 5 - Figure Supplement 1

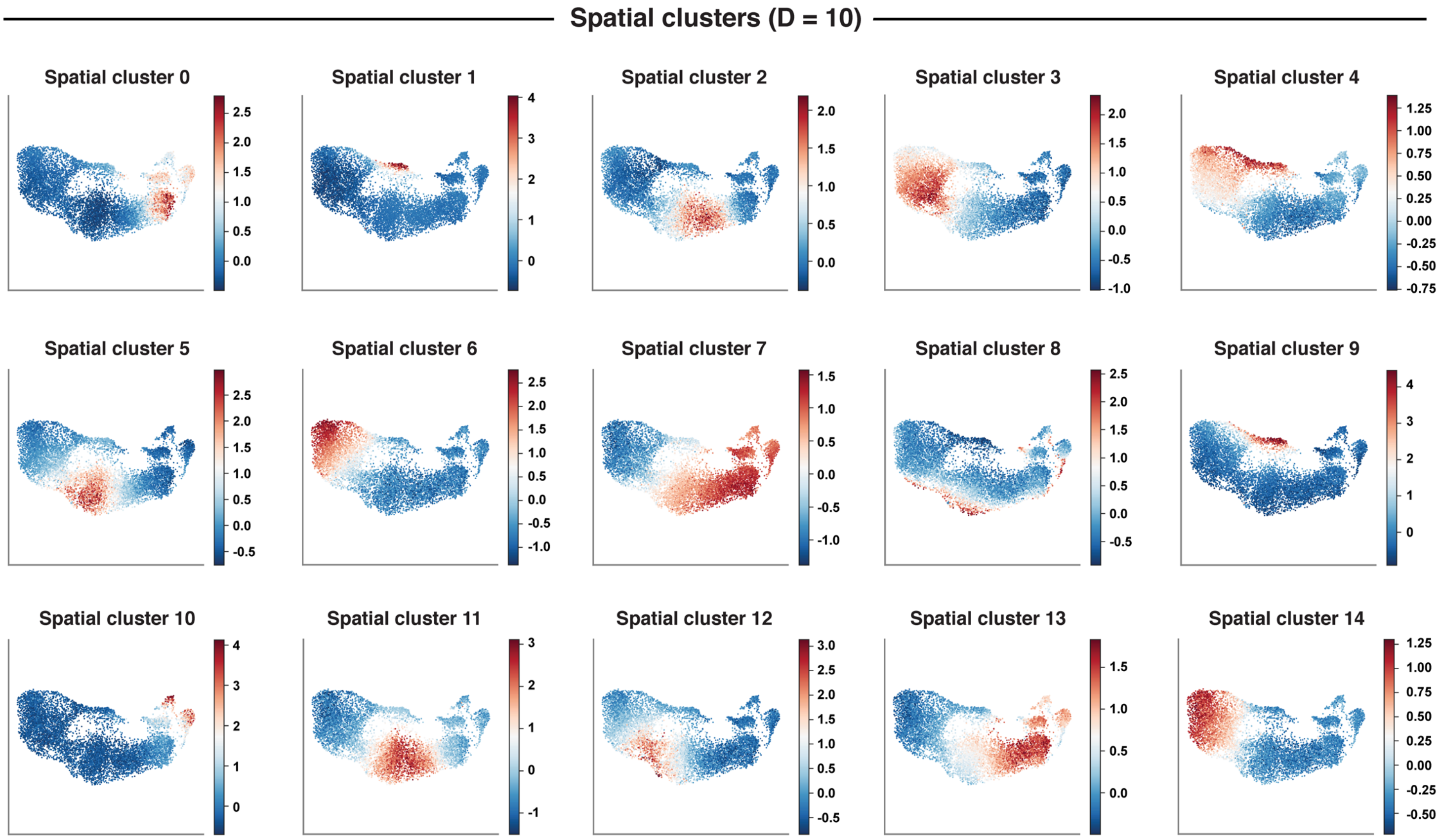

Figure 6 - Figure Supplement 1

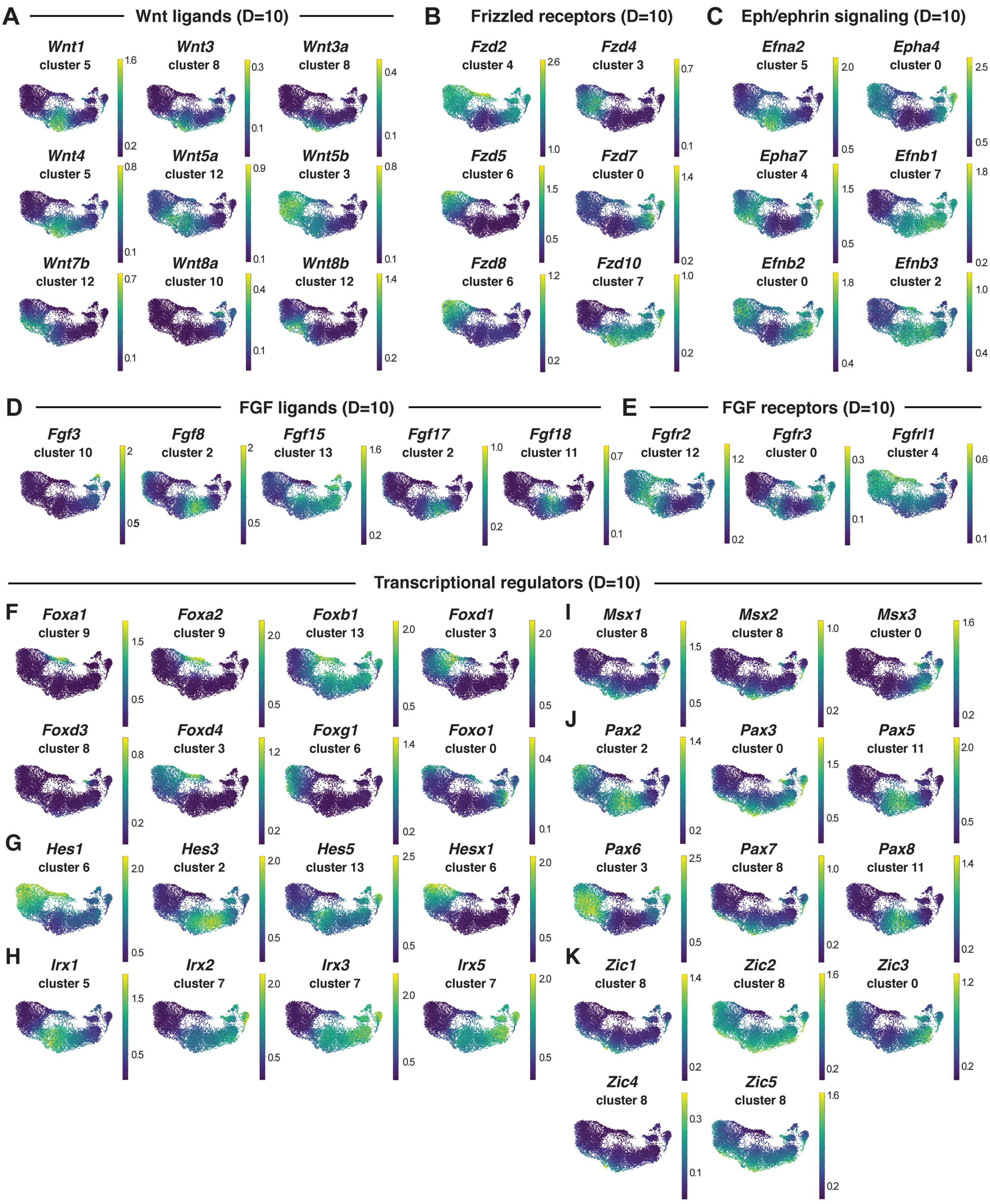

Supplement: Supplementary file 2 [file media-1.pdf]
